# Effects of sildenafil and tadalafil on skin flap viability

**DOI:** 10.1007/s00403-021-02196-0

**Published:** 2021-03-14

**Authors:** Rafael A. C. Souza, Carla Patrícia Martinelli-Kläy, Armando J. d’Acampora, Geraldo J. S. Bernardes, Sandro M. Sgrott, Laila A. C. Souza, Tommaso Lombardi, Thaís R. Sudbrack

**Affiliations:** 1 Federal University of Santa Catarina, Florianópolis, Brazil; 2 Santa Casa de Taquaritinga Hospital, Taquaritinga, Brazil; 3 Laboratory of Oral and Maxillofacial Pathology, Oral Medicine and Oral and Maxillofacial Pathology Unit, Rue Michel-Servet 1, 1211 Geneva 4, Switzerland; 4 University of Southern Santa Catarina, Florianópolis, Brazil

**Keywords:** Flap viability, Type-5 phosphodiesterase inhibitor, Healing

## Abstract

Vascular complication is one of the causes of skin flap healing failure. Sildenafil and tadalafil, a type-5 phosphodiesterase inhibitor, can improve flap viability, however, the action mechanisms involved in this process are still unclear. To assess the effects of orally administered sildenafil and tadalafil on the healing kinetics and skin flap viability, sixty-two Wistar rats were divided into three groups: control (*n* = 22), sildenafil (*n* = 20), and tadalafil (*n* = 20). The solutions were administered orally (dose: 10 mg/kg) immediately after the surgical procedure and then every 24 h. At postoperative days 7 and 14, the skin flap samples were collected, submitted to histological processing and evaluated under optical microscopy. In experimental groups (sildenafil and tadalafil), we found an increased vascularization (*p* < 0.05) on the 7th and 14th day associated with the ulcer size decrease on the 14th day, although it was not significant. There was a higher influx of neutrophils and a decrease of mononuclear population on the 7th day (*p* < 0.05). On the 14th day, these differences were observed only in the tadalafil group (*p* < 0.05). This study suggested positive results with the use of sildenafil and tadalafil as adjuvant drugs in skin flap viability.

## Introduction

In healing, one of the critical events is angiogenesis, essential for the re-establishment of blood flow and oxygen support to injured tissues [1].

A flap is a unit of tissue that can be transferred from donor to recipient sites, maintaining its blood supply during the process [2]. Despite attempts to transfer tissue without damaging the blood supply, flaps may show signs of poor perfusion and venous congestion in sectioned vascular structures. Besides the complexity of the scarring process, flaps undergo a crucial phase in their viability due to the inherent changes during tissue transfer [3].

The NO pathway starts with NO inactivating the enzyme, guanylate cyclase, resulting in increased levels of cGMP and leading to smooth muscle relaxation in blood vessels. Phosphodiesterase-5 (PDE5) inhibitors improve the vasodilatory effect of NO through cyclic guanosine monophosphate (cGMP) [4]. Phosphodiesterase-5 inhibitors have beneficial effects in angiogenesis, endothelial proliferation, remodeling, and improvement of the oxygen supply during healing [5–8]. They can regulate vasodilation [9], clear oxidative stress components [10] and present some antimicrobial activities [11]. The commercially available PDE-5 inhibitors are sildenafil (Viagra), vardenafil (Levitra), tadalafil (Cialis) and udenafil (Zydena).

Sildenafil was initially developed as an antihypertensive drug. Considering its vasodilatory effect, its current characteristic indications, include sexual dysfunction and pulmonary hypertension. Other benefits from sildenafil-induced vasodilation have been investigated, including studies on the improvement of flap viability, colonic anastomosis, pressure ulcers, fractures, microvascular anastomoses, musculoskeletal injury, and scarring [12–22]. Despite the number of studies on sildenafil application (most studied drug of all drugs of this class) to increase flap viability, some pharmacological features indicate that tadalafil has the highest applicability in random skin flaps [23]. These PDE5 inhibitors have prolonged half-lives with sildenafil (4 h), vardenafil (4–5 h), udenafil (12 h), and tadalafil (17.5 h), which facilitate a longer effect without the inconvenience and side effects of multiple doses [23].

Several studies have been conducted to clarify the exact effects of PDE5 inhibitors on wound healing and flap viability. Although sildenafil remains the drug of primary interest in this class, other drugs including tadalafil have been studied with relative success [23–27]. Nonetheless, the results observed in the literature are conflicting and the action mechanisms involved in this process are still unclear [13, 15, 23, 26, 28, 29, 30, 31].

## Materials and methods

### Animals

All the procedures were performed according to the current guidelines for experiments involving animal models, namely, the Brazilian Federal Law No. 11,794/2008 and 2016 Guidelines for the Care and Use of Animals for Scientific and Teaching Purposes from the Brazilian National Council for the Control of Experimentation with Animals (CONCEA). The research protocol was approved by the University of Southern Santa Catarina’s Committee of Ethics in the Use of Animals (No. 16.016.4.01.IV). We used male Wistar rats (*n* = 62; age: 60 days; mean weight: 350 g) at different observation periods (7 and 14 days). The animals submitted to stress or discomfort and the ones that evolved to death during any phase of the experiment were excluded from the study. The animals were divided into three groups depending on the agent used: (1) sildenafil group: sildenafil citrate; (2) tadalafil group: tadalafil; and (3) control group: a syrup vehicle containing sucrose. In all groups, the solutions were administered in a solution form using an orogastric tube (dose: 10 mg/kg per animal) immediately after the surgical procedure and every 24 h. The groups were assessed at postoperative days 7 and 14, and each group was medicated daily until the end of the experiment.

### Experimental procedure

Anesthesia was performed using 5% and 2% aqueous solutions of S-(+)-ketamine hydrochloride (75 mg/kg) and xylazine hydrochloride (10 mg/kg), respectively, administered via a deep intramuscular injection internally into the left hind limb (quadriceps femoris) [32, 33].

When reaching the anesthesia plane, the animals were placed in the ventral decubitus position on wooden plates (30 cm × 35 cm) and fixed with strings on the front (*membrum thoracicum*) and hind (*membrum pelvinum*) legs. After epilation and antisepsis, a rectangular area (2 cm × 6 cm) was longitudinally marked with a skin marker pen conventionally used in plastic surgeries, considering the upper scapular region as the base and the spine as the central reference.

**Fig. 1:**
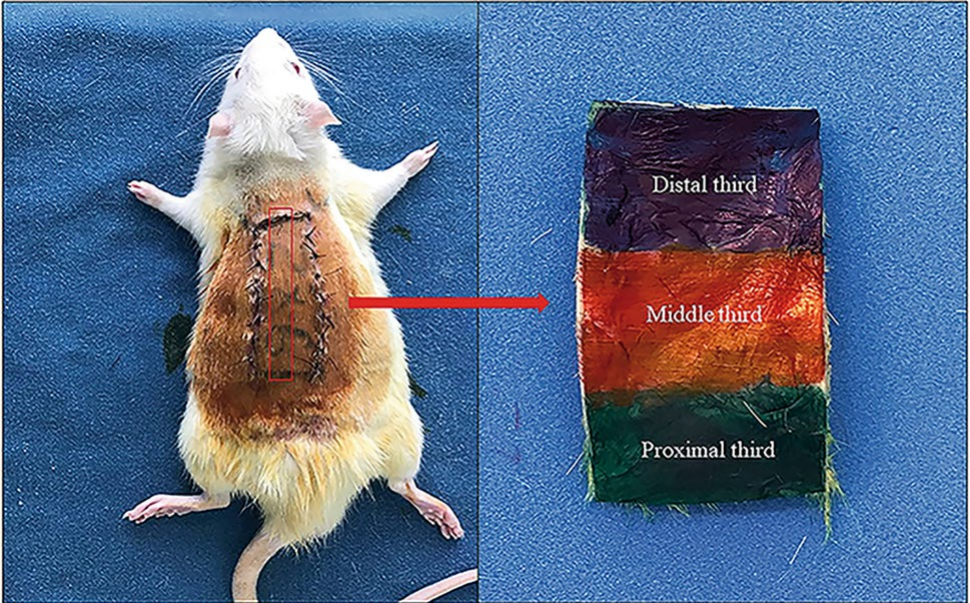
Flap segment used for histological evaluation

The markings were incised with a 15-blade scalpel, and the flap skin was detached from the dorsal musculature; immediately thereafter, the flap was repositioned on the receptor bed and sutured using nylon 4.0 (Mononylon^®^, Ethicon, São Paulo, Brazil), with single sutures at a 0.5 cm of distance from each other, securing the flap skin in its original position (Fig. 1).

After anesthetic recovery, the animals were retained at the Laboratory of Operative Technique and Experimental Surgery at room temperature, with continuous airflow and free of noise and stress, following the natural day and night cycles. The animals were placed in individual numbered cages, placed on shelves at an equal distance from the light source, and received Nuvilab Cr-1 feed and water ad libitum. For postoperative analgesia, metamizole was added to drinking water at approximate doses of 150–600 mg/kg (considering the mean water intake by weight of the species) for seven consecutive postoperative days. No animals were lost during the study. Adverse effects to medication as bleeding were not observed.

Following euthanasia, samples were collected and examined in accordance with the guidelines of the CONCEA (NR37) and Brazilian Federal Council of Veterinary Medicine (Resolution 1000/2012).

### Histological analysis

The collected samples were immediately fixed in 10% buffered formalin, submitted for routine histological processing with hematoxylin and eosin (H&E) staining, and analyzed by a pathologist (double-blinded design). Regarding the inflammatory process, the percentage of neutrophils and mononuclear cells was calculated at the proximal junction from the green to the red zone (Fig. 1) in three 400 × contiguous fields. Neovascularization was evaluated by counting vascular lumens in three contiguous 400 × fields at the proximal junction. The histological analysis of the inflammatory cells and blood vessels was not performed at the distal portion because of the variable presence of inflammation and necrosis. The size of the true (bloody) ulcers observed below the scab was analyzed. All ulcer measurements were performed in the middle portion of the flap (Fig. 1).

### Statistical analysis

Statistical analysis was performed using the statistical program SPSS (version 17; IBM, Armonk, NY, USA). After performing Levene test, ANOVA was used to evaluate the vascularization variable. For the other variables, the Kruskal–Wallis test was used. For all statistical tests, a 5% level was considered statistically significant [34].

## Results

**Fig. 2:**
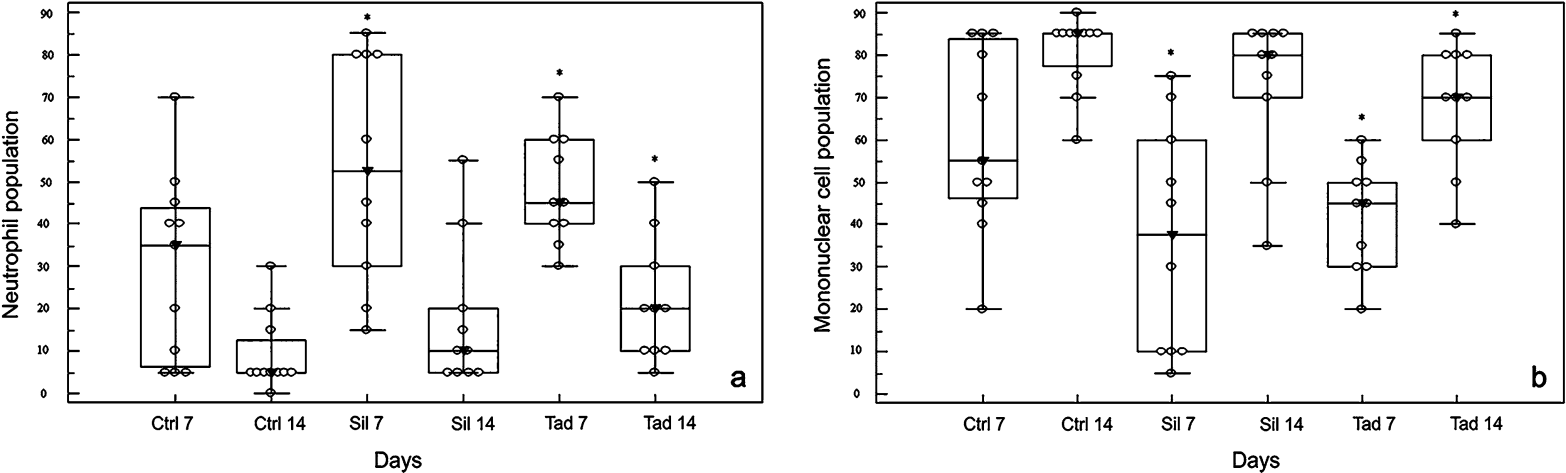
Distribution of groups at postoperative days 7 and 14 according to neutrophil cell (**a**) and mononuclear cell percentages (**b**). Empty circles indicate the observed cases; medians are indicated by a filled inverted arrowhead. Ctrl 7 (control 7 days), Ctrl 14 (control 7 days), Sil 7 (sildenafil 7 days), Sil 14 (sildenafil 14 days), Tad 7 (tadalafil 7 days), Tad 14 (tadalafil 14 days). *p < 0.05

The neutrophils population on the 7th day after the surgery was significantly higher in the experimental groups (sildenafil and tadalafil) when compared to the control group (*p* < 0.05). The medians of these cell percentages observed in the control, sildenafil and tadalafil groups were 35.0 (IQR = 6.0–44.0%), 52.5 (IQR = 30.0–80.0%), and 45.0 (IQR = 40.0–60.0%), respectively (Fig. 2a). On postoperative day 14, these values decreased in all groups: control, 5.00 (IQR = 5.0–12.0%); sildenafil, 10.00 (IQR = 5.0–20.0%), and tadalafil, 20.00 (IQR = 10.0–30.0%). However, the neutrophil population remained higher in the experimental groups when compared to the control group, although only the administration of tadalafil presented a statistically significant difference compared to the control group (*p* < 0.05). The difference between the medians of the experimental groups was not statistically significant (*p* > 0.05). Regarding the mononuclear cell population, a significant increase (*p* < 0.05) was observed from 7 to 14th day in all groups, which is consistent with the tissue repair kinetics. However, on the 7th postoperative day (Fig. 2b), there was a smaller mononuclear cell population in the experimental groups when compared to control group (*p* < 0.05). The medians of mononuclear cell percentages of the control, sildenafil, and tadalafil groups were 65.0% (IQR = 6.0–44.0%), 47.5% (IQR = 20.0–70.0%), and 55.0% (IQR = 40.0–60.0%), respectively. At day 14, significant differences were observed only between the control and tadalafil groups (*p* < 0.05). The control group had a median of 95.0% (IQR = 88.0–95.0%) vs. 90.0% (IQR = 80.0–95.0%) in the sildenafil group and 80.0% (IQR = 70.0–90.0%) in the tadalafil group.

**Fig. 3:**
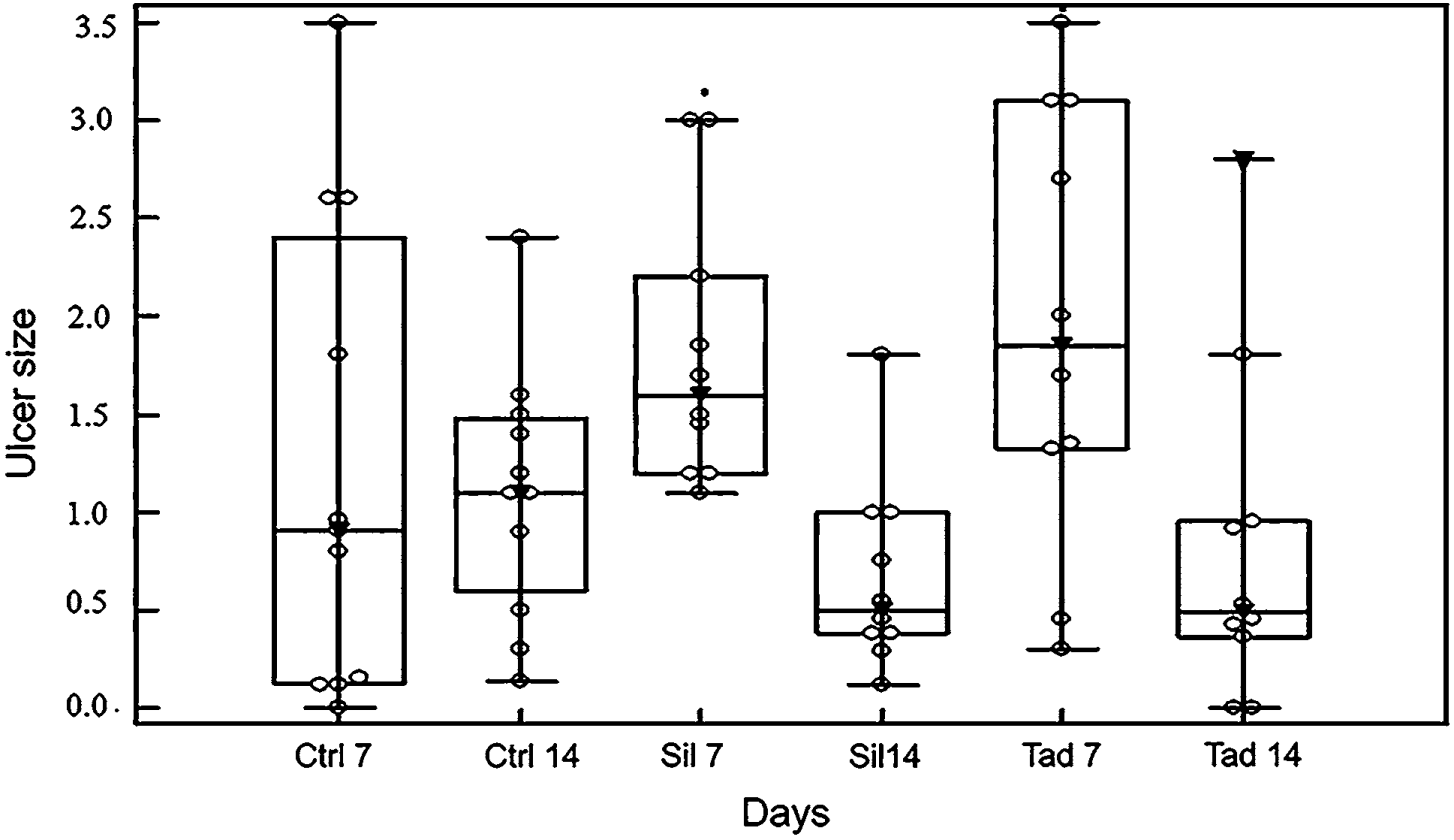
Distribution of groups at postoperative days 7 and 14 according to median ulcer size. Empty circles indicate the observed cases; medians are indicated by a filled inverted arrowhead. Ctrl 7 (control 7 days), Ctrl 14 (control 7 days), Sil 7 (sildenafil 7 days), Sil 14 (sildenafil 14 days), Tad 7 (tadalafil 7 days), Tad 14 (tadalafil 14 days). *p < 0.05

The size pattern of the true ulcerated region (bloody tissue) under the visible tissue scab (or ulcer) is shown in Fig. 3. At postoperative day 7, there was a statistically significant increase in ulcer size in the experimental groups, when compared to the control group (*p* < 0.05). The median of the control group was 0.91 cm (IQR = 0.13–2.40) vs. 1.6 cm (IQR = 1.20–2.20) and 1.85 cm (IQR = 1.32–3.10) in the sildenafil and tadalafil groups, respectively. From the 7th to the 14th day, a considerable decrease (*p* < 0.05) in the size of the ulcers in all groups was observed. On the 14th day, the ulcers of the experimental groups were lower than the control group, although this difference was not significant. The median was 1.1 cm (IIQ = 0.60–1.48) for the control group, against 0.50 cm (IIQ = 0.38–1.00) for the sildenafil group and 0.49 cm (IIQ = 0.36–0.95) for the tadalafil group.

**Fig. 4:**
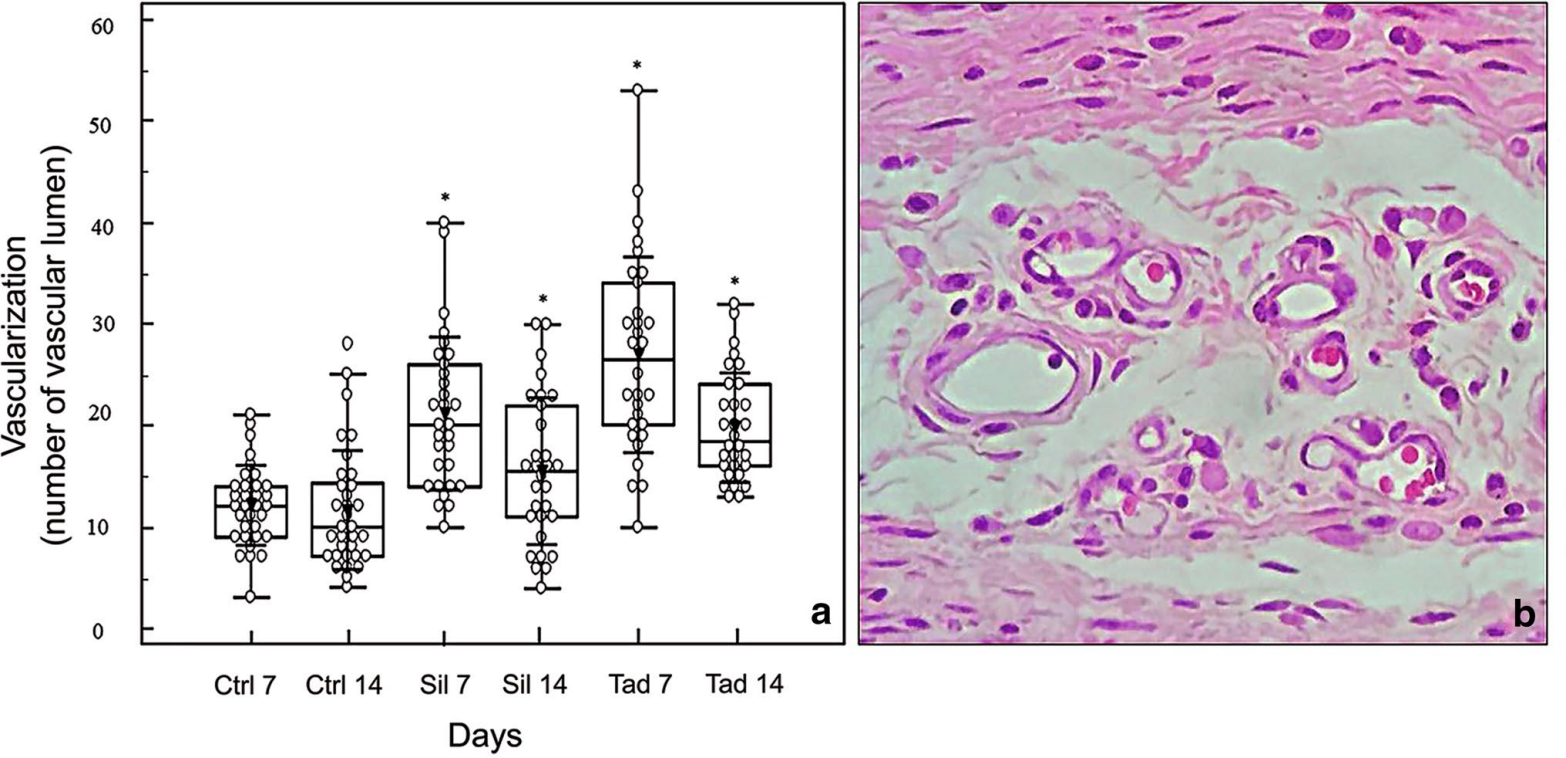
Distribution of groups at postoperative days 7 and 14 according to mean vascularization. Empty circles indicate the observed cases, means are indicated by a filled inverted arrowhead, and error bars indicate ± 1 standard deviation (4a). Histological feature of vascular lumens (blood vessels, 4b). Ctr 7 (control 7 days), Ctr 14 (control 7 days), Sil 7 (sildenafil 7 days), Sil 14 (sildenafil 14 days), Tad 7 (taladafil 7 days), Tad 14 (taladafil 14 days). *p < 0.05

The pattern of the vascularization variable described in Fig. 4 showed the differences in terms of lumen count between the experimental group and the control group. At postoperative day 7, while the median in the control, sildenafil, and tadalafil groups was 11.4196 ± 3.92, 19.9129 ± 7.54, and 25.2461 ± 9.68, respectively, at postoperative day 14, these values decreased to 10.3668 ± 5.90, 13.7915 ± 7.29, and 19.2121 ± 5.36 in the control, sildenafil, and tadalafil groups, respectively. However, the number of blood vessels was higher in the experimental groups when compared to the control group on both 7th and 14th day (*p* < 0.05).

## Discussion

Random skin flaps are often used in wound reconstructions and coverage. They are mostly used as a resource in plastic surgeries to repair substance losses. Resection due to oncological treatment, decubitus ulcers, and trauma often cause loss of skin and subcutaneous tissue, resulting in aesthetic and functional defects. The skin barrier deficiency can then lead to infection or tissue loss. In addition, wound treatments are expensive for the healthcare system and an important public health issue [24, 35].

Flap extent is often limited by its perfusion, particularly in its distal portion. Partial or total loss of this flap remain a vital problem in reconstructive surgeries. Total flap necrosis is rare, but can cause a great increase in morbidity [36]. In this respect, several medications have demonstrated positive effects on healing and flap viability. Among these, PED-5 inhibitors (e.g., sildenafil and tadalafil) appears as strong candidates considering their mechanism of action [8].

Despite the clinical application in an empirical way, there are few publications that report the use of five phosphodiesterase inhibitors to improve the skin flap viability [37].

It is known that PED-5 inhibitors improve the vasodilatory effect of NO through cyclic guanosine monophosphate (cGMP) [4]. Accumulation of cGMP promotes smooth muscle relaxation and consequently an increase of the blood flow in target organs [38]. Besides the use of PDE-5 inhibitors to treat erectile dysfunction, investigators are now proposing novel and off-label uses for these agents such as pulmonary hypertension. Approval from the Food and Drug Administration has now been granted to use PDE-5 for the treatment of pulmonary hypertension [8]. Flushing, headache, and dizziness are, however, the most prevalent side-effects of sildenafil. Hemoptysis and hemorrhagic stroke are other important adverse effects [39–41]. Indeed, studies have shown that sildenafil may inhibit collagen and ADP-induced platelet aggregation ex vivo [41]. In the current study, we do not report hemorrhagic events.

We used sildenafil and tadalafil in the concentration of 10 mg/kg per animal. After 7 and 14 postoperative days the healing kinetics was analyzed. In the literature, most animal studies assess postoperative day 7 due to strong evidence of the drug action in the three initial days of healing [23, 24, 28, 35, 38]. However, little is known about the healing kinetics on the 14th day, which is vital for random flap viability and better represents the so-called proliferative stage [42]. With respect to drug concentration, the vast majority of studies adopted a dose of 10 mg/kg/day as a point of convergence for sildenafil and tadalafil [23, 24, 28, 35, 38]. Sarifakioğluet al. [38] also suggested a dose-dependent effect (3, 10, and 20 mg/kg), in which a progressive dose increase improves the flap viability.

The result analysis of neutrophils and mononuclear cells on the 7th and 14th day is based on the pharmacokinetics of the referred drug class. With vasodilation, an increase of neutrophil concentration is observed in the acute phase [42, 43]. PDE5 inhibitors potentiate this vasodilatory effect [11, 44] and would probably increase the inflow of these cells in the initial and late period of the flat healing. Furthermore, the prolonged action of Tadalafil could be responsible for maintaining the high level of neutrophils in the period of 14 days, since the influx of neutrophils, although not significant, was greater in the tadalafil group than in the sildenafil group. This increase of neutrophils in the treated groups may explain how these drugs affect wound healing. A study performed in animal models submitted to median laparotomy [15] showed a significantly higher neutrophil population in the sildenafil group when compared to the control group on the 14th day. However, Choi et al. [29] presented, although not statistically significant, a lower influx of neutrophils in skin flap of rats submitted to treatment with sildenafil (20 mg/kg/day, enteral) when compared to alprostadil (prostaglandin E1) within a 7-day follow-up period. On the 7th day Kaya et al. [26] did not find any significant differences of neutrophils, in the skin flaps of the mice treated for 3 days with sildenafil, tadalafil and vardenafil. In a study wherein the effects of tadalafil (5 mg/kg/day, 4 days, orally) on the healing of ischemic small intestine anastomoses were evaluated in a rat model, no differences were observed between the control and tadalafil groups regarding inflammatory infiltration. [30] According to Szczypka and Obmińska-Mrukowicz [45], the peritoneal macrophages treated with sildenafil, produced a greater amount of interleukin beta (IL-1 β) and NO causing an increase in the percentage of phagocytosing granulocytes and a decrease in phagocytosing monocytes. Previously considered only responsible for the first line of local defense, neutrophils can also help in the resolution of inflammation and repair activation. New studies have pointed out to a change in the role of neutrophils in healing, promoting vascularization. Oxidants and proteases, considered “toxic” substances produced by neutrophils, may have a healing function. It is likely that there are subtypes of neutrophils, which would explain this dichotomy of beneficial and harmful roles of neutrophils. In this context, we must not forget that several studies demonstrate that neutropenic patients suffer from scar delay [46, 47].

In our study the results also indicated, although not significant, a reduction of the ulcerated region in the experimental groups on the 14th day. It indicates a better flap evolution and reduction of necrosis in the experimental groups compared to the control group. However, on the 7th day, an increase in the ulcer size of the treated groups was observed in relation to the control group (*p* < 0.05). In 2011, Barral et al. [13] found, on the 7th day, an increase in flap necrosis in the group of rats submitted to treatment with subdermal sildenafil, at a dose of 0.5 mg/kg/day for 3 days. In contrast, Kaya et al. [26] observed macroscopically a non-significant decrease in the necrosis of the skin flaps of rats treated orally (10 mg/kg/day, for 3 days) with sildenafil, tadalafil and vardenafil. Contrarily, a study [23] evaluating axial flaps, showed a significant macroscopic reduction of necrosis in rats treated with injectable tadalafil for 3 days. These variations regarding the ulcer size may be the result of the different methodology used.

Regarding vascularization, the “expected” effect of PED5 inhibitors on flaps was observed in our study, with increased vascular lumen count in the experimental groups, particularly in the tadalafil group. Local blood supply is essential for the entire healing process. Considering the mechanism of action presented by this class of drugs [8, 14, 16, 17, 48], a positive effect was expected at some stage of the scarring process. Blood inflow is not only necessary to supply the recovering wound but also responsible for carrying the first repair and defense cells to the repair site [42, 43]. In a study (2005), fibrin glue was used as the delivery medium for sildenafil, which was applied locally under the flap. After 7 days, the histological samples were evaluated and a significant increase in microvascular density was observed among the groups treated with the assessed drug [31]. On the other hand, in a study (2008) comparing the effects of sildenafil and VEGF, combined or not, a statistically insignificant increase in longitudinal vascular density was observed only in the VEGF-treated group [28]. Tadalafil also increased the intestinal rupture pressure and the hydroxyproline concentration in normal and ischemic anastomoses in rats orally treated with 5 mg/kg/day, during 4 days. However, no differences were observed between the groups regarding vascularization or deposition of collagen in anastomosis [30]. According to the findings of the present study, the kinetics of these drugs can effectively alter the healing and viability of skin flaps in animal models through vascular, cellular, and possibly humoral factors. Understanding these mechanisms may help to reach a better view of the scarring process in this drug class.

## Conclusion

This result evidenced the effect of the drugs on increased vascularization during the two observation periods, associated with an increase in the influx of neutrophils in 7 and 14 days. Although not statistically significant, a reduction of ulcer size on postoperative day 14 was also observed. The present data are important because they provide further evidence for the clinical applicability of PDE5 inhibitors on flap healing and viability. Nevertheless, further studies are needed to better evaluate the mechanism of action of these drugs.
